# Precaution or barrier? Reconsidering contact and isolation measures in palliative care: a systematic scoping review

**DOI:** 10.1017/ash.2025.10096

**Published:** 2025-08-07

**Authors:** Henry T. He, Shannon Bunn, Brittany Rance

**Affiliations:** Division of Palliative Care, University of British Columbia, Vancouver, BC, Canada

## Abstract

**Objective:**

Infection control measures like contact precautions may conflict with patient-centered palliative care principles, but their efficacy and harms in this context remain understudied. This review evaluates how contact precautions affect quality of life, social connectedness, and infection control efficacy in palliative care.

**Design:**

Systematic scoping review.

**Setting:**

Palliative care settings (eg, palliative care units and hospices)

**Participants:**

Adults and children receiving palliative care, with no restrictions on age or comorbidity.

**Methods:**

English-language studies on contact precautions in palliative care were included. Ovid MEDLINE and Ovid Embase were searched from inception to December 20, 2024, using terms related to antimicrobial resistance, contact precautions, and palliative care. No publication type or status restrictions were applied. The protocol was registered on the Open Science Framework and followed Preferred Reporting Items for Systematic reviews and Meta-Analyses extension for Scoping Reviews guidelines.

**Results:**

Fifteen studies were included, primarily from Germany (73%) and using qualitative methods (80%). Most focused on patients in palliative care units or hospices, though geographic and methodological limitations restrict generalizability. Common challenges included fear, loneliness, disrupted intimacy, and inconsistent protocols. Contact precautions were often bundled with other infection prevention interventions, limiting the ability to assess their specific impact. Terminology varied widely. No study directly evaluated the efficacy of contact precautions in reducing antimicrobial-resistant organism (ARO) transmission, though one pediatric study reported liberal protocols and no nosocomial ARO infections.

**Conclusions:**

A case-by-case approach is needed to balance infection control with patient dignity and quality of life. Consistent terminology and more robust, mixed-methods research are essential to inform evidence-based protocols in diverse settings.

## Introduction

Infection and colonization with antibiotic-resistant organisms (AROs)—also known as multidrug-resistant organisms (MDROs) or organisms which develop antimicrobial resistance—is known to be associated with negative patient outcomes including longer hospital lengths of stay and higher mortality.^1^ The most studied organisms include methicillin-resistant *Staphylococcus aureus* (MRSA), vancomycin-resistant *enterococci* (VRE), and gram-negative bacteria such as extended-spectrum β-lactamase producing *Enterobacterales* (ESBL-E). The prevalence of antimicrobial-resistant organisms (AROs) also continues to grow, partly due to antibiotic overuse, increased morbidity of chronic disease associated with lower immune status, and the widespread use of indwelling medical devices.^2^ In the hospice and palliative care unit (PCU) setting, the prevalence of MRSA colonization, for example, is reported in the range of 3%–11.6%.^2–5^ PCUs are typically inpatient units integrated into hospitals, where hospital admission criteria apply. These units may not always be closed, meaning that patients admitted under other medical services—such as internal medicine or surgery—may also be cared for within the same unit. Hospices, by contrast, are usually freestanding facilities that focus primarily on nursing and comfort care for patients at the end of life.

Infection prevention and control protocols typically employ a multifaceted bundled approach to control transmission of AROs, including antibiotic stewardship, active surveillance, contact or barrier precautions, environmental decontamination, and decolonization.^6^ Contact precautions (CPs) are one method used to prevent transmission of AROs, and typically involve the isolation of patients in private rooms and using physical barriers (gowns and gloves) for all healthcare personnel and visitors when entering the room as defined by the Centers for Disease Control,^7^ however may also be facility-specific. They are widely used despite limited evidence supporting their efficacy from a small number of cluster-randomized clinical trials and systematic reviews in the acute non-palliative setting.^6,8–12^ The evidence base for CPs is often limited by a focus on outbreak scenarios causing performance bias, selection bias toward specific AROs (eg, MRSA, VRE), failure to study CPs independently from bundled Infection prevention and control interventions, inadequate compliance monitoring, and underrepresented infection risk factors (eg, immunocompromised status and indwelling devices).^12^ CPs are also known to impose significant burdens and harms for patients, including social isolation, delays in care, and reduced interaction with healthcare workers, as well as additional financial costs to the healthcare system.^2,13–15^ These issues are very relevant in palliative care, where patient comfort and dignity are paramount.

The objective of this scoping review is to characterize the effectiveness of contact precautions alone against transmission of AROs in adult and pediatric patients receiving palliative care in any care setting, in addition to patient-centered outcomes including quality of life and social connectedness. A scoping review approach is ideal for synthesizing diverse evidence, identifying knowledge gaps, and guiding future research to align CP protocols with the principles of palliative care.

## Methods

### Protocol and registration

The protocol for this scoping review is registered on the Open Science Framework. It can be accessed at the following link: https://doi.org/10.17605/OSF.IO/TV3KC. The Preferred Reporting Items for Systematic reviews and Meta-Analyses extension for Scoping Reviews (PRISMA-ScR) guideline was followed during the article writing phase, and the completed checklist can be found in Appendix A within the Supplemental Materials.^16^

### Eligibility criteria

The eligibility criteria for this scoping review include studies published in English, with no restrictions on publication type or status, that explore the impact of CPs on both adult and pediatric patients receiving palliative care. There were no restrictions on medical comorbidities.

### Search strategy

The search was conducted in Ovid MEDLINE (1946–December 20, 2024) and Ovid Embase (1974–December 20, 2024) databases with the help of a university librarian, using a combination of subject heading terms and keywords such as “antimicrobial drug resistance,” “contact precautions,” and “palliative care,” targeting study titles, abstracts, and keywords. Detailed electronic search strategies can be found in Appendix B within the Supplemental Materials.

### Screening process

**Figure 1.**
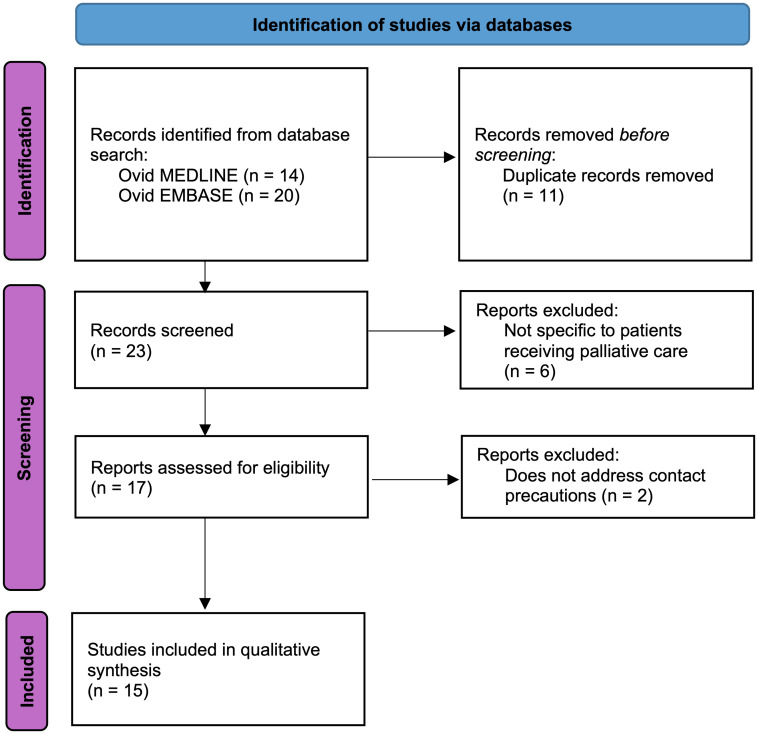
PRISMA flow diagram. Template source: *Page MJ, et al BMJ 2021;372:n71. doi: 10.1136/bmj.n71*.

Studies were screened by the primary author (HH) to determine whether they met the inclusion and exclusion criteria. All reasons for exclusion were recorded. Once included, a full text review conducted by the primary author (HH) identified study characteristics including study design, aim, population, comparative groups, relevant findings, and limitations. A PRISMA flow diagram is shown in Figure 1 documenting the selection process.

### Data items

Extracted data items included the first author and year of citation, the country of the study, study design (including methodology and period), and population characteristics. Key findings were recorded, focusing on infection control outcomes, patient experience, and quality of care, along with study limitations such as small sample sizes, single-site data, or any potential biases. Assumptions included considering studies mentioning contact precautions in palliative or end-of-life care as relevant, treating synonymous terminology (eg, “barrier precautions”) as interchangeable, and interpreting findings across healthcare systems while acknowledging policy variations. Simple percentages were used to report study characteristics without any formal statistical analysis, as the focus was on mapping existing literature rather than drawing inferential conclusions.

## Results

The search strategy described in the Methods returned 34 publications (Ovid MEDLINE: 14, Ovid EMBASE: 20). After 11 duplicate studies were excluded, 23 studies underwent title and abstract screening. Of these, 6 studies did not meet the inclusion criteria as they were not specific to patients receiving palliative care.^17–22^ The remaining 17 studies underwent full-text review, and 2 studies were excluded as they did not address contact precautions.^4,23^

Fifteen studies were ultimately included in this review,^24–38^ with details summarized in Table 1. A notable proportion of the included studies (11 out of 15, 73%) were conducted in Germany. Of these, 8 were part of the “MRSA in End-of-Life Care” (M-EndoL) interdisciplinary project, funded by the German Federal Ministry of Education and Research^26,28,30,32–36^ and two focused on the “PALLINI” hygiene concept for pediatric patients.^37,38^ Only one review article originated from North America.^27^ The majority of studies (12/15, 80%) employed qualitative or mixed-methods designs, primarily using questionnaires or interviews, alongside a descriptive case report,^25^ a review article,^27^ and an observational surveillance study.^38^ Fourteen studies (93%) involved patients in adult or pediatric PCUs or hospices, with half also including geriatric ward patients. One study lacked clarity regarding the setting,^29^ and three were project abstracts with limited details available.^26,29,31^

**Table 1.** Study characteristics

First author y, country	Study design (period)	Aim	Population	Relevant findings	Limitations
Bükki 2013, Germany^24^	Questionnaire-based descriptive qualitative study (January 2009 to February 2009)	To explore the current practice of MRSA management and its impact on inpatients’ quality of life as perceived by professional caregivers	All PCUs and hospices listed in Germany’s directory of palliative care services (n = 360)	• Over 90% of responding institutions had specific MRSA protocols, but resource constraints, such as single-room availability (*P* = .002) and staffing (*P* = .004), were more significant for PCUs compared to hospices • PCUs were more likely than hospices to isolate MRSA patients (*P* = .000), treat colonization (*P* = .026), assess eradication efficacy (*P* = .000), and restrict patient activities (*P* = .000) • MRSA protocols in PCUs were associated with a higher reported negative impact on patient quality of life (*P* = .000) compared to hospices	• Self-reporting bias • Only members of the professional teams were approached, not patients or family members • Clinical roles or professions of respondents are unknown • Only MRSA was studied
Cheng 2014, Hong Kong SAR, China^25^	Descriptive case report (July 2013)	To discuss a clinical case involving the ethical dilemma of balancing patient safety while avoiding social isolation of the dying	A PCU in Hong Kong	• There is insufficient data on VRE colonization in palliative care despite known patient risk factors and higher complication rates of VRE infections in this population • Large-scale studies on VRE prevalence, the impact of infection control measures on patient and family quality of life, and outbreak prevention guidelines for multidrug-resistant organisms in palliative care is a “high research priority.”	• Descriptive case report on a single patient; no data collection or analysis collected • Only VRE was studied
Sturm 2016, Germany^26^	Interview-based qualitative study (duration unknown). Part of M-EndoL*	To explore the impact of MRB on the patient experience regarding information, communication, ailments, therapy and contact precautions	Patients admitted to geriatric and palliative wards (n = 43)	• MRB impacts patients differently, affecting physical health, social life, and emotional state • Adaptation varied based on health status, primary diagnosis, MRB history, and family situation, and reflect diverse personal experiences	• Abstract from a poster presentation; full study details unavailable
Datta 2017, USA^27^	Descriptive Review Article	To review the burden and management of MDROs in palliative care	Patients receiving palliative care	• Bacterial infections impact over one-third of advanced cancer or terminal illness patients, significantly increasing mortality. Differentiating colonization from infection is challenging in palliative care due to cognitive impairment, metastatic disease, and atypical symptoms • Antimicrobial treatment may lack symptom relief and can be invasive, costly, or burdensome. While MDROs like MRSA are highly transmissible and linked to worse outcomes compared to MSSA, colonization in nonsterile sites or asymptomatic cases may not require treatment • MRSA prevalence in palliative care (9–12% in PCUs, 4–8% in hospices) is lower than in intensive care units (20%) or nursing homes (up to 50%), with VRE and ESBL-E rates estimated at 10–33% and 15–20%, respectively. Contact precautions often conflict with palliative care principles, causing distress and complicating bereavement, leading to calls for their removal • Infection management should align with goals of care, weighing potential symptom relief against the burdens of testing, treatment, and complications	• Lack of systematic search strategy
Heckel 2017, Germany^28^	Interview-based qualitative study (April 2014 to September 2015). Part of M-EndoL*	To assess the effects of the patient’s MRSA/MRGN colonization or infection and isolation measures on family caregivers.	Family caregivers of patients with MRSA or MRGN from a PCU, a hospital palliative care support team, and a geriatric ward (n = 62)	• Family caregivers stressed the need for clear, consistent communication on MRSA/MRGN and hygiene measures. • They voiced concerns about stigma and disadvantages tied to the diagnosis, and psychosocial and emotional support was a key priority	• Only MRSA and MRGN was studied • Also included geriatric patient population (21/62 participants)
Schunck 2017, Brazil^29^	Mixed methods study (January 2015 to December 2016)	To evaluate the infection rates of a Hospital for Palliative Care and to verify the incidence of death related to hospital infection	Private Hospital for Palliative Care in São Paulo, Brazil	• Infection incidence ranged from 4.99 to 5.85 per 1 000 patient-days, with death rates of .23 to .61 per 1 000 patient-days, aligning with international data for long-stay institutions • The most common infections were urinary tract infections, pneumonia, gastrointestinal infections, and integumentary infections, with varying incidence rates across years	• Abstract from a poster presentation; full study details unavailable • Unclear admission criteria or patient demographics • Bundled infection prevention and control approach included surveillance, outbreak control, and isolation precautions
Heckel 2017, Germany^30^	Interview-based qualitative study (March 2014 to December 2014). Part of M-EndoL*	To gather institutional stakeholders’ perspectives on MDROs in end-of-life care and inform recommendations for managing and implementing isolation measures	Institutional stakeholders (n = 18) with leading positions in the fields of clinic, nursing, hygiene, infection control, administration, and management at two study centers: a German PCU and a geriatric ward	• Institutional stakeholders noted the tension between infection control and patient-centered care in palliative and geriatric settings and faced a dilemma between protecting others and preserving patients’ quality of life • In the absence of clear guidelines, they suggest a case-based approach	• Generalisability of findings limited due to small sample size (two centers in Germany)
Myat Aye 2018, Australia^31^	Questionnaire-based qualitative study (June 2014 to August 2014; September 2016 to November 2016)	To explore patient, family, and staff perceptions of contact precautions in palliative care, with a focus on emotional impact	Distributed to patients, relatives and staff in the PCU at Bethesda Hospital in Perth (n unknown)	• Most patients (55%), relatives (82%), and staff (89%) viewed contact precautions as necessary in palliative care • Despite this, 67% of staff reported a perceived negative impact of contact precautions on patient care, and 56% noted a negative impact on families • Both patients and families acknowledged the negative effect of contact precautions on patient well-being (36% respectively)	• Abstract from a poster presentation; full study details unavailable • Unclear total number of participants, patient demographics or clinical roles of staff
Tiedtke 2018, Germany^32^	Interview-based qualitative study (March 2014 to February 2015). Part of M-EndoL*	To explore healthcare professionals’ experiences, feelings and attitudes about caring for hospitalized patients with multidrug-resistant bacteria in palliative and geriatric care	35 staff members from a German PCU and a geriatric unit (nurses, physicians, psychologists, social workers, other therapeutic professions, and volunteers)	• Team members reported increased workload, disrupted routines, and tension between palliative care and infection control • Balancing patient care and contamination prevention led to “unsolvable conflicts,” ambivalence, and varied coping strategies	• Generalisability of findings limited for other care settings (ie hospices)
Heckel 2018, Germany^33^	Mixed-methods study (March 2014 to April 2016). Part of M-EndoL*	To provide empirical recommendations on how to deal with hospitalized MDRO patients in end-of-life care	Recommendations were based on 158 interviews, six focus groups (n = 39), and input from 17 experts	• 21 recommendations support a case-based approach to MDRO management at end of life • Key points include admission screening, resource-aware accommodation, and prioritizing social inclusion • Emphasis was placed on clear patient-family communication, transparent documentation, and staff training	• Limited generalisability due to small sample size (two German centers)
Peters 2019, Germany^34^	Retrospective analysis of M-EndoL* qualitative interview data using linguistic methods	To replicate and complement previous findings using a different methodological framework (linguistic model)	Transcribed interviews with family caregivers in a palliative care setting (n = 50)	• Caregivers’ communication varies by involvement level and stance toward staff and the hospital, forming four profiles: passive-cooperative (trusting), passive-confrontational (resigned), active-cooperative (egalitarian), and active-confrontation (aggressive) • Each profile requires tailored staff communication (written and spoken), although clear, family-centered dialogue benefits all	• Lack of phonetic transcription may have limited analysis of prosody, emphasis, and pauses, which provide contextual cues for caregiver attitudes • While the proposed categories covered most cases (94%), some interviews did not fit, requiring a larger data set for broader applicability
Heckel 2020, Germany^35^	Mixed methods study (March 2014 to September 2015; February 2016). Part of M-EndoL*	To explore patients’ perspectives on MDRO management, its impact on well-being, and their care preferences	Interviews with MDRO-positive patients (n = 43) in a German PCU and geriatric ward, plus a focus group (n = 8) with geriatric patients, families, and staff	• Patients in end-of-life and geriatric care experience significant emotional and social impacts due to multidrug-resistant bacterial diagnoses, stringent hygiene measures, and insufficient information, affecting their quality of life • Focus group discussions highlighted patients’ desire for clearer communication, comprehensive information, and reduced social isolation	• Limited generalisability due to small sample size (two German centers) • Quantitative study component was not feasible due to missing data
Heckel 2020, Germany^36^	Mixed methods study (June 2019). Part of M-EndoL*	To evaluate the implementation status of national recommendations on managing MDRO in end-of-life care published in 2017	Staff members on the PCU at a university hospital (n = 20)	• Most staff (18/20) knew the national recommendations; after one year, 12 of 27 were highly implemented, and 13 were partly integrated into daily routines. • Two recommendations had low implementation: (i) accounting for additional time constraints due to protection/isolation measures in personnel and bed planning, and (ii) facilitating patient recognition of team members and family caregivers • Improvements were reported postimplementation, but barriers included procedural complexity, poor checklist usability, and behavioral/cognitive issues like transmission-related anxiety and over-reliance on protective clothing. Enhancing the checklist, simplifying procedures, and embedding practices into daily routines were identified as key next steps	• Lack of patient and family caregiver perspectives in the study •Staff satisfaction with caring for MDRO patients was not assessed beyond implementation status
Schmidt 2021, Germany^37^	Cross-sectional mixed-methods study (February 2018 to January 2020)	To identify the impact of a complex hygiene concept (“PALLINI”) and its core component barrier nursing on children’s and parents’ quality of life and social participation	Interviews were conducted with parents and staff at a PPCU in a tertiary children’s hospital (n = 47), and parent-proxy questionnaires assessing the quality of life of children were also collected (n = 163)	• In pediatric patients with life-limiting conditions and MDRO colonization, complex hygiene protocols and barrier nursing facilitated social participation, but also created barriers including stigmatization, fear of contamination and doubts regarding safety • The child’s quality of life did not appear to be significantly affected	• Limited generalisability due to small sample size (single German center) • Quantitative results interpretated with caution due to large variation between comparison group sample sizes
Schmidt 2022, Germany^38^	Observational surveillance study (February 1, 2018 to January 31, 2020)	The study aimed to assess MDRO prevalence, the predictive value of risk factors, and the incidence of nosocomial infections and colonisations on a PPCU implementing the “PALLINI” hygiene concept, with barrier nursing as its core component.	Patients admitted to the PPCU in a tertiary care children’s hospital (n = 165) who were screened for MDRO	• The MDRO colonization rate at admission was 12.7%, with prior positive MDRO screening identified as the only significant individual risk factor • Over two years, no MDRO-related nosocomial infections were reported, and the incidence density of nosocomial colonization was .6, suggesting that the risk-adapted barrier-nursing-based hygiene concept (PALLINI) was safe	• Family members and hospital staff were not screened • Over two years, only 3/165 patients with negative MDRO screening at admission were colonized at discharge. Genetic analysis indicated nosocomial transmission was unlikely, suggesting false-negative initial screens due to natural colorectal colonization

Abbreviations: *M-EndoL, “MRSA in End-of-Life Care” interdisciplinary project funded by the Federal Ministry of Education and Research; MRSA, methicillin-resistant *Staphylococcus aureus;* MSSA, methicillin-sensitive *Staphylococcus aureus;* VRE, vancomycin-resistant *Enterococci;* MRB, multidrug-resistant bacteria; MRGN, multi-resistant gram-negative bacteria; MDRO, multidrug-resistant organism; PCU, palliative care unit; PPCU, pediatric palliative care unit.

### Contextualizing ARO prevalence in palliative care settings

Although most included studies did not directly assess the prevalence of AROs, a few provided contextual data relevant to understanding the broader landscape of ARO exposure in palliative care. For example, Datta and Juthani-Mehta noted that bacterial infections affect over one-third of patients with advanced cancer or terminal illness and are associated with increased mortality, although differentiating colonization from infection is often challenging in this population due to cognitive impairment, metastatic disease, and atypical symptoms.^27^ While not the primary focus of this review, limited prevalence estimates were available: MRSA prevalence ranged from 9%–12% in PCUs and 4%–8% in hospices, which was lower than in intensive care units (20%) and nursing homes (up to 50%).^27^ VRE and ESBL-E prevalence rates were estimated at 10%–33% and 15%–20%, respectively.^27^ In pediatric palliative care units, Schmidt et al reported a 12.7% ARO colonization rate at admission.^37^

### Definitions of contact precautions

Definitions of contact precautions in the literature are inconsistent, and this variation in terminology was reflected in the included studies. For example, the M-EndoL study described precautions and isolation measures as “all measures applied with the intention to avoid transmission and spreading of MDRO in hospitals.” These included personal protective equipment (eg, gloves, eye protection, protective clothing, caps, filtering facepiece respirators), cleansing agents, disinfectants, single-room accommodation, and hand hygiene.^28,33,35,36^ In the PALLINI studies, a “barrier nursing-based hygiene concept” was described to involve strict barrier nursing practices, the use of gowns, rigorous hand disinfection, and maintaining physical distance (1–1.5 m) from others during group activities such as music therapy or communal meals. Inside patient rooms, doors could remain open, and personal protective equipment was required only for staff during close contact—not for patients or their family members.^37,38^ Other studies did not provide explicit definitions and used various terms, including “close contact isolation,”^25^ “patient isolation and contact precautions,”^27^ “contact precautions,”^26,31^ “isolation measures,”^32,34^ and “isolation precautions.”^29^

### Prevalence of contact precautions

While most studies reported bundled approaches, Bükki et al categorized individual MRSA management policies from 229 German PCUs and hospices, and reported their prevalence.^24^ In total, over 90% of PCUs and hospices had specific MRSA protocols. Isolation of MRSA-positive patients was reported by 99% of PCUs and 76% of hospices, while activity restrictions were noted by 96% of PCUs and 66% of hospices. Common precautionary measures included the use of gloves, gowns, face masks, handwashing, and hand disinfection. Mandatory precautions for staff were reported by 99% of PCUs and 100% of hospices, and recommended precautions for visitors were reported by 98% of both PCUs and hospices.^24^

### Efficacy of contact precautions

The included studies did not directly assess the efficacy of contact precautions in reducing the transmission of AROs. However, one observational surveillance study conducted in a pediatric PCU in Germany reported no ARO-related nosocomial infections over a two-year period following the implementation of a “barrier nursing-based hygiene concept”.^38^ Of 165 patients with negative ARO screening at admission, three were found to be colonized at discharge (one with MRSA, one with VRE, and one with multidrug-resistant *Escherichia coli*). Whole-genome sequencing indicated no close genetic relatedness between isolates, suggesting that nosocomial transmission was unlikely and that initial false-negative screening results may have been responsible.

### Perceptions on contact precautions

Myat Aye and Bulsara reported that while most patients (55%), relatives (82%), and staff (89%) viewed CPs as necessary in palliative care, significant concerns about their impact were noted.^31^ Among staff, 67% reported a perceived negative effect on patient care, and 56% noted a negative impact on families. Similarly, both patients and families acknowledged the adverse effects of CPs on patient well-being (36% respectively).^31^

### Impact of AROs and contact precautions

Bükki et al reported that compared to hospices, PCUs more frequently isolated patients with MRSA and restricted their activities, which was associated with a higher negative impact on patient quality of life.^24^

Heckel’s M-EndoL project revealed the profound impact of AROs on patients, family caregivers, healthcare providers, and institutional stakeholders through rich interview data.^26,28,30,32,35^ Patients on CPs for AROs expressed ignorance *(“I do not know anything about the germ, except that I have it”*), indifference *(“so many other fears are dominant for me—the cancer diagnosis. That is why the germ does not bother me much”*), and fear *(“The [MDRO] takes my life away”*), with isolation exacerbating loneliness (“*You feel like a leper*”) and disrupting intimacy (“*We did not touch each other anymore. No kisses. That was not nice*”).^35^ Family caregivers faced confusion over inconsistent protocols (“*One time he had to wear a protective garment, the next time he didn’t have to. I didn’t really know what was going on*”) and emotional strain, with some prioritizing mental well-being (“*I don’t want to know too much about MRSA. I have formed a concept, and I am fairly okay with it*”).^28^ Peters et al conducted a linguistic analysis of these caregiver interviews and concluded that caregivers’ communication varies by involvement and stance, and formed four profiles: passive-cooperative (trusting), passive-confrontational (resigned), active-cooperative (egalitarian), and active-confrontational (aggressive).^34^ Each profile required tailored staff communication; however clear, family-centered dialogue benefitted all.

Healthcare providers grappled with balancing infection control and compassion, noting the emotional toll of precautions *(“You can touch him, but with gloves it’s something completely different. That’s an additional constraint which isn’t nice, especially at the end of life*”) and the need for individualized care (“*I think an individual approach is the best we can offer these patients”*).^32^ Interdisciplinary team members reported increased workload, disrupted routines, and tension between palliative care and infection control, leading to “*unsolvable conflicts,”* ambivalence, and varied coping strategies. Institutional stakeholders emphasized the tension between emotional needs (“*There are issues such as talking to the family, relationships, interpersonal closeness and so forth that play a completely different role*”) and strict measures (“*To me it is important that everyone sticks to the isolation measures which are based on scientific findings*”), advocating for flexibility (“*Sometimes I rather get a phone call asking, ‘How can I handle this situation correctly?’ instead of adhering to the guidelines*”).^30^

### Recommendations for managing AROs in palliative care

The included studies suggest several recommendations for managing AROs in palliative care. Datta and Juthani-Mehta’s review highlighted the conflict between CPs and palliative care principles, which can cause distress and complicate bereavement, prompting calls for their removal and advocating for infection management aligned with goals of care.^27^ For example, antimicrobial treatment for AROs like MRSA may lack symptom relief and can be invasive or burdensome, suggesting that colonization in nonsterile sites or asymptomatic cases may not always require treatment. Heckel et al developed 21 recommendations for managing AROs in end-of-life care, grounded in qualitative data and expert consensus, with an overarching emphasis on case-based application of protection and isolation measures.^33^ Implementation data of these recommendations on a national level in Germany showed awareness and at least partial integration of most of these recommendations after one year, however isolation-related time constraints, procedural complexity and transmission anxiety were identified as persistent challenges.^36^ In the pediatric setting, Schmidt et al found that a more liberal barrier nursing focused hygiene protocol enabled social participation but introduced challenges such as stigmatization and safety concerns, and did not significantly impact quality of life.^38^

## Discussion

To the authors’ knowledge, this is the first systematic scoping review to examine the use of CPs to prevent ARO transmission and infection in the palliative care setting. The findings of this review highlight the complex interplay between infection control measures and palliative care principles. Balancing the need to reduce morbidity and mortality from ARO infections with the potential harms of infection control interventions—such as CPs, which can increase social isolation and emotional distress for patients, families, and healthcare professionals—presents a significant challenge. This complexity is further compounded by resource limitations and the varied responses and experiences of all stakeholders. The literature generally recommends a case-based approach to ARO management including the use of CPs, emphasizing clear communication and staff training. However, research in this area remains limited, with most studies originating from Germany, which restricts the generalizability of the findings.

This review has several limitations. The small number of studies included in the review and difficulty in identifying unpublished studies or institutional policies precluded us from an assessment of publication bias. The decision to include only English-language studies was due to limited resources for professional translation, which may have led to the exclusion of relevant studies published in other languages. Additionally, variations in terminology and reporting styles across studies may have affected data extraction and synthesis. The absence of formal quality assessment or risk of bias, as is standard in scoping reviews, means that the strength of the evidence could not be critically appraised. Finally, geographic representation was limited, making it unclear how findings translate to different healthcare systems and cultural contexts.

While this review aimed to specifically identify contact precautions, most included studies described bundled Infection prevention and control interventions that combined contact precautions with other measures such as patient isolation, hand hygiene, and environmental disinfection. This reflects a common challenge in infection prevention and control research, where interventions are implemented as multifaceted bundles, making it difficult to isolate the effects of individual components. Further complicating this is the inconsistent and interchangeable use of terminology across studies, with terms such as “barrier precautions,” “isolation measures,” and “contact precautions” often used non-specifically. Notably, none of the included studies directly evaluated the efficacy of contact precautions in reducing the transmission of AROs, despite this being one of the stated objectives of the review. However, one observational surveillance study in a pediatric PCU suggested that implementing a more liberal contact and isolation protocol was associated with no observed nosocomial ARO infections or colonizations.

Further research is needed to inform evidence-based CP protocols in palliative care, with attention to patient quality of life. Mixed-methods studies may offer valuable insights by integrating infection control outcomes with the perspectives of patients, caregivers, and healthcare providers. Locally conducted research can support the development of context-specific policies that address both safety and care values. Future work that combines epidemiological evidence with lived experiences may contribute to CP protocols that are clinically appropriate while also minimizing potential harms.

## Supporting information

10.1017/ash.2025.10096.sm001 He et al. supplementary material He et al. supplementary material

## Supplementary material

The supplementary material for this article can be found at https://doi.org/10.1017/ash.2025.10096.
